# A QUALITATIVE STUDY ON THE COGNITION AND EXPERIENCES MOBILITY DEVICES USE IN KNEE OSTEOARTHRITIS PATIENTS OF CHINA

**DOI:** 10.1093/geroni/igz038.3086

**Published:** 2019-11-08

**Authors:** Fumin Dai, Mengdie Jiang, Wenwen Jia

**Affiliations:** 1 Henan Provincial People’s Hospital, Zhengzhou, Henan province, China; 2 Henan University, Zhengzhou, Henan, China

## Abstract

The benefits of mobility devices for knee Osteoarthritis Patients include reducing burden of knee joint, enhancing confidence and increasing autonomy, yet many who might benefit from using mobility devices do not use them. The purpose of this study is to explore the cognition and using experiences about mobility devices use in patients with knee osteoarthritis of China. Naturalistic inquiry research was adopted, 15 patients with knee osteoarthritis were recruited and Semi-structured interviews were conducted. Data were analyzed based on conventional content analysis methodology. Two themes of using experiences about mobility devices were extracted, including positive feelings, negative feelings. Cognition about mobility devices use included light consciousness, incorrect attitude. Mobility devices have a positive effect on knee osteoarthritis patients, but some patients lack sufficient understanding and face many problems in the use of mobility devices. Policy Support, greater physician involvement, positive peer modules, and safe, visually appealing devices would promote greater acceptance and satisfaction of mobility devices with knee osteoarthritis patients.

